# Contrasting Changes in Strongly and Weakly Bound Hydration Water of a Protein upon Denaturation

**DOI:** 10.1021/acs.jpcb.3c02970

**Published:** 2023-07-07

**Authors:** Mafumi Hishida, Ayumi Kaneko, Yasuhisa Yamamura, Kazuya Saito

**Affiliations:** † Department of Chemistry, Faculty of Science, Tokyo University of Science, 1-3 Kagurazaka, Shinjuku, Tokyo 162-8601, Japan; ‡ Department of Chemistry, Faculty of Pure and Applied Sciences, University of Tsukuba, Tsukuba, Ibaraki 305-8571, Japan

## Abstract

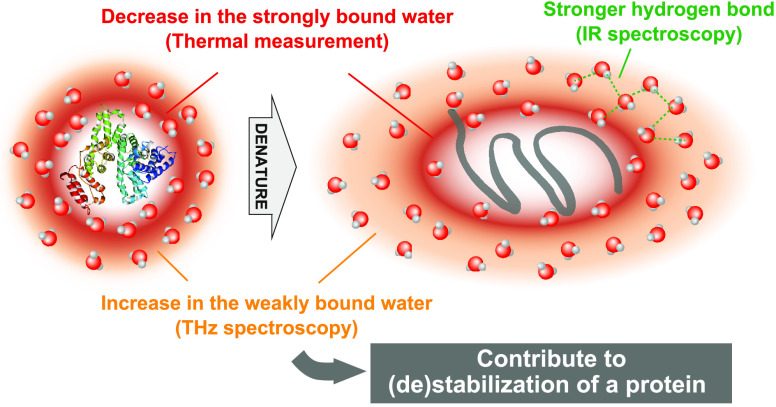

Water is considered integral for the stabilization and function of proteins, which has recently attracted significant attention. However, the microscopic aspects of water ranging up to the second hydration shell, including strongly and weakly bound water at the sub-nanometer scale, are not yet well understood. Here, we combined terahertz spectroscopy, thermal measurements, and infrared spectroscopy to clarify how the strongly and weakly bound hydration water changes upon protein denaturation. With denaturation, that is, the exposure of hydrophobic groups in water and entanglement of hydrophilic groups, the number of strongly bound hydration water decreased, while the number of weakly bound hydration water increased. Even though the constraint of water due to hydrophobic hydration is weak, it extends to the second hydration shell as it is caused by the strengthening of hydrogen bonds between water molecules, which is likely the key microscopic mechanism for the destabilization of the native state due to hydration.

## Introduction

Water is the most universal and abundant substance on Earth, and its properties are strongly related to the functionalities of various materials and living cells. However, many of its fundamental physical properties remain unclear. Hydration is one of the most important issues in solving the relationship between (bio)materials and water. A hydration water layer is formed in the region surrounding a dispersed solute, and many studies have been conducted on the hydration states of materials dispersed in water.^1−5^ Hydration has also recently become a hot topic for research as it is actively involved in the functionality of materials and development of biological phenomena.^6−14^ For example, cooperative dynamics between proteins and water play an important role in the physiological activity of proteins.^10,11^ However, the overall picture of hydration remains unclear, and an improved understanding from the micro to the macro scale is urgently required.

Protein stability is one of the most important issues related to hydration and is an active research subject.^11−14^ Numerous proteins exhibit their functions by folding into specific structures in their native state. However, they can also be denatured by heat and acid treatments, which leads to the unfolding of the structures and exposes their hydrophobic groups to water.^15−19^ It is important to understand the mechanisms that maintain the folded structure in the native state, from the viewpoints of both life sciences and materials sciences. From the perspective of thermodynamics, it has been shown that the Gibbs energy of the protein solution is strongly affected by hydration water.^20−26^ The difference in the Gibbs energy between the native and denatured states of proteins without water is much larger than that in aqueous solutions. This indicates that hydration water destabilizes the folded structure of the proteins. The energetic balance between the native and denatured states should be discussed, considering not only the differences in the higher-order structure of the polypeptide chains but also the differences in hydration between these states. Recently, it has been revealed that entropy in terms of the state of water is larger in the native state Crambin solution (−*T*Δ*S* ≈ 500 kJ/mol) than in the denatured state, while the entropy of protein itself favors the unfolded denatured state.^26^ Hydration water is also known to cause cold denaturation.^27,28^

Thermodynamics for the hydration states (i.e., macroscopic descriptions) of proteins under denaturation has been widely studied. However, we do not fully understand the microscopic changes of hydration water under denaturation, although the microscopic aspect of the hydration state has been widely investigated only in the native state.^11,12,14,29−34^ Model proteins such as bovine serum albumin (BSA), which is used in this paper, have been used to show that some water molecules are directly bound at various sites and that weakly bound water exists over long distances in the native state.^29−32^ This long-range hydration layer fluctuates, and a collective hydrogen-bond network reconstructs over a wide time range of 1–200 ps depending on the surface site of the protein. The dynamics of this time range are thought to strongly correlate with protein structuring and chemical properties.^34^ This long-range hydration is likely related to protein denaturation. Some hydrophobic groups are exposed to water due to protein denaturation, and change in the state of the surrounding water is suggested.^35−37^ However, the details of this change are unclear (e.g., changes in the hydration number, the hydrogen-bond structure, and the mobility of water in the hydration layer). The microscopic mechanism underlying the role of water on protein stability against denaturation and how it relates to the macroscopic thermodynamics of hydration are not well understood. Integrated information on hydration changes upon denaturation at the microscopic scale, that is, how the structure, dynamics, and number of hydration water change with denaturation, is thus required to understand protein stabilization mechanisms in detail.

Along with protein stabilization, studying the hydration of solutes at the microscopic scale has faced numerous other problems. One is that the relationship between the information obtained using various methods to observe hydration is unclear. Hydration can be observed by focusing on various physical properties using methods such as thermal measurements,^4,38,39^ nuclear magnetic resonance (NMR) spectroscopy,^29,30,40^ neutron scattering,^11,12,14,41^ dielectric relaxation spectroscopy,^9,32,42^ terahertz spectroscopy,^31,32,35,45−47^ and infrared spectroscopy.^33,43,44^ However, because the physical quantities observed by these methods are essentially different, whether the observed hydration water is the same and the relationship between the physical quantities is unclear. Therefore, basic information such as the number of hydration water varies widely between reports, making it difficult to obtain a complete picture.

Another problem derived from the lack of standardization of the observed information is that the definition of the phenomenon of hydration is vague. In the simplest definition, hydration water can be defined as a water molecule whose motion is bound through interactions such as hydrogen bonds and electrostatic interaction with a solute. However, since the degree of water binding varies with the surface functional groups of the protein and its distance from the surface, the number of hydration water varies greatly depending on what degree of binding is considered hydration water. It has been proposed that strongly bound water exists on the surface of the solute (mainly in the first hydration shell), whereas weakly bound water exists outside of it (mainly in the second hydration shell);^12,48^ however, the exact definition of each remains unclear. In addition, in some cases, the acceleration of water mobility by hydration has been reported.^49,50^

The challenges related to solving the above problems have led to the question, “What is hydration?” To answer this, it is necessary to observe the hydration of a unique sample using various methods and integrate the observed information to understand the relationship between each physical quantity. To clarify the relationship between the information obtained from these various methods, it is valuable to compare not only the identified numbers of hydration water but also the responses to external stimuli, such as temperature changes. That is, heat-induced protein denaturation is one of the best research topics.

For an integrated understanding of hydration, it is essential to observe the weakly bound hydration water, which has rarely been observed, and understand its relationship with the state of the strongly bound hydration water, mainly in the first hydration shell. For this purpose, we used terahertz (THz) spectroscopy,^51^ which is one of the most important methods used in this study. The dynamics of water molecules or their clusters can be observed in the terahertz frequency range.^45−47^ As described below, the hydration state can be determined by the changes in the motion of molecular dynamics.^49,52−54,57^ For example, the application of terahertz spectroscopy to the phospholipid bilayer shows a hydration layer with bound motions of 4–5 layers (∼28 water molecules per phospholipid molecule) on the membrane surface.^53^ In contrast, NMR and neutron scattering observations of water motion in the slower frequency range have estimated only a single hydration layer on the surface (approximately six molecules per phospholipid molecule).^58,59^ This implies that only strongly bound water is observed as hydration water in the latter, whereas the hydration in the former includes weakly affected water molecules. This was also confirmed for proteins, where hydration over several layers was observed by THz spectroscopy.^31,32,60^ When using these methods, hydration water is defined as water whose molecular mobility, such as diffusion, is altered by the solute. However, thermal measurements and infrared spectroscopy, which are the methods used in this study, have completely different definitions of hydration. In thermal measurements, the water that does not freeze on cooling (unfreezable water or nonfreezing water) is quantified.^4,38,39^ This layer is thought to be strongly influenced by the surface. When using infrared (IR) spectroscopy, the hydration state is defined by the frequency change of the OH stretching vibration. Water molecules whose hydrogen bonds are strengthened because of the solute affection are usually regarded as hydration water using this method.^33,60^ The relationships between the molecular dynamics, antifreeze behavior, and OH vibrations of water are not well understood.

In this study, we used complementary methods to integrate microscopic information relating to the hydration of proteins and investigate changes in the state of water during the denaturation of proteins and the underlying causes. THz spectroscopy, infrared spectroscopy, and thermal measurements were used to observe the changes in the hydration state of BSA upon denaturation by heating. Importantly, we found that the response of the hydration information to denaturation was completely different depending on the observation method, indicating that the strongly and weakly bound hydration water had different responses to the denaturation. These results help to elucidate the energetic balance between native and denatured proteins, including the contribution of hydration water.

## Methods

### Samples

BSA (FUJIFILM Wako Pure Chemical, >95%) was used as the model protein without further purification. BSA was mixed with ultrapure water (MilliQ, 18.2 MΩ·cm) at 13 wt %. The solution was clear and homogeneous. The density of the BSA solution was measured using a density meter (DMA35, Anton Paar) for temperatures < 40 °C, and for temperatures > 40 °C, extrapolation was utilized (see the results in the Supporting Information). The volume fraction of BSA in solution was obtained from the density and then used for THz spectroscopy analysis.

### Terahertz Time-Domain Spectroscopy

THz spectroscopy was performed using custom-made terahertz time-domain spectroscopy (THz-TDS) equipment.^49^ An ultrafast pulse fiber laser (FemtoFErb780, TOPTICA, 780 nm, 100 fs, 50 MHz) was used as the infrared (IR) light source. The optical length of one of the split IR lights was changed using a delay stage. The emission and detection of the THz waves were performed using dipole photoconductive antennas (SD-TX101 and SD-RX101, Pioneer). After narrowing the THz wave to several millimeters in diameter with a hyperhemispheric lens, it was focused on the sample position using plastic lenses, and lock-in detection was then performed. The entire system, including the fiber laser, was purged with dry air (QD-20-50 and RD-45-N, IAC. Co., Ltd.). At the sample position, we applied an attenuated total reflection (ATR) setup to accurately determine the complex dielectric function of aqueous solutions using a Dove prism made of silicon (refractive index of 3.4). The design of the ATR prism was the same as previously described.^52,61^ At the prism surface, the polarization of the THz wave was set to p-polarized to detect even a small change in the dielectric constant induced by the hydration effect of the solute. The penetration depth of the evanescent field of the THz wave was ∼20 μm. The temperature of the ATR sample cell was controlled using a Peltier device with proportional-integral-derivative (PID) control (TDC-1010A, Cell System Co., Ltd.). With our setup, the complex dielectric constants could be precisely determined with high reliability in the 0.3–2.5 THz regions. The measurements of pure water were repeated six times at the same temperature. The measurements of BSA solution were repeated six times (<60 °C) or four times (>60 °C). For the fitting analysis, the averaged data and its standard deviations were used.

### Differential Scanning Calorimetry

Differential scanning calorimetry (DSC) was performed using commercial equipment (Q200, TA instruments). The temperature of the equipment was calibrated using indium (156.6 °C) and pure water (0 °C), and the heat flow was calibrated using the melting enthalpy of frozen pure water. The temperature range was −75 to 85 °C. First, we confirmed whether there was no thermal anomaly between −75 and 0 °C, as these would indicate that the sample did not include the so-called “intermediate water”^4,39^ that freezes at a much lower temperature than 0 °C. Only “unfreezable water (nonfreezing water)” that does not freeze by cooling^4,38,39^ was measurable with this method. To investigate the number of unfreezable water, the following temperature cycle was employed with a ramp rate of 2 °C/min: (1) cooled from room temperature (21–23 °C) down to −25 °C, (2) heated from −25 °C up to an arbitrary temperature *T*_0_, (3) cooled from *T*_0_ to −25 °C, and (4) heated from −25 °C to *T*_0_. From the thermal anomaly corresponding to the melting of water during cycles (2) and (4), the number of unfreezable water was determined. The measurements were repeated four times at the same conditions, and their averages and standard deviations were used for the analysis.

### Fourier-Transform Infrared Spectroscopy

Fourier-transform infrared (FTIR) spectroscopy was conducted using commercial equipment (FT/IR-4200, JASCO) with an ATR setup (ATR PRO450-S, JASCO). The temperature was controlled between 35 and 75 °C using a heater attachment (PHE-600 and TC-300, JASCO). A total of 5 μL of sample solution was dropped onto the ATR prism. The number of accumulations was set to 64, and the averaged spectra were used for analysis. The fitting error was used for the error bars in the ratio of three components of OH bands.

### Circular Dichroic Spectroscopy

Circular dichroic (CD) spectra were measured between 190 and 250 nm using commercial equipment (J-820, JASCO). The temperature was controlled using a Peltier device (CDF-426L, JASCO). For the CD spectrum measurements, a 13 wt % BSA solution was diluted 10,000 times. The optical length of the quartz cell was 1 cm. The number of accumulations was set to 3, and the averaged spectra were used for analysis.

## Results

### Denaturation of BSA

**Figure 1:**
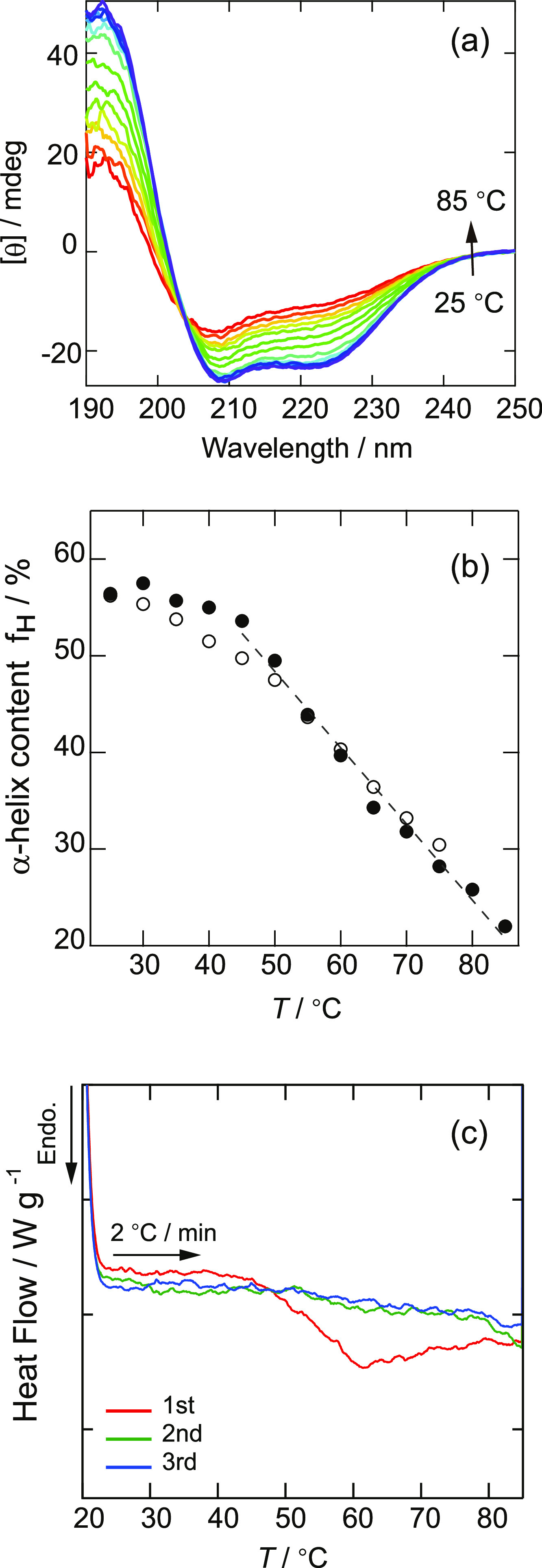
Determination of denaturation temperature of BSA by CD spectroscopy and DSC. (a) CD spectra for BSA solution at each temperature. [θ] is the averaged residual molar ellipticity. (b) α-helix fraction *f*_H_ in BSA before (●) and after (○) freezing. The dashed line represents the linear fitting result for *f*_H_ before freezing. (c) DSC curves for BSA solution between 25 and 85 °C.

The results of circular dichroism (CD) spectra and DSC measurements for the denaturation behavior of BSA are shown in Figure 1. Figure 1a shows the temperature dependence of the CD spectrum of BSA in pure water. Using the ellipticity at 222 nm, [θ]_222_/°, the α-helix fraction *f*_H_ in BSA was calculated using the following equations^62^
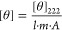
1
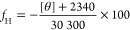
2where [θ] is the average residual molar ellipticity, *l* is the optical path length (1 cm), *m* is the molar concentration of BSA (=2.05 × 10^–7^ mol/L), and *A* is the number of amino acid residues in BSA (583). The results for *f*_H_ presented in Figure 1b showed little difference regardless of the freezing procedure, indicating that freezing does not largely change the secondary structure of BSA. Previous reports have shown that the α-helix content of BSA decreases from ∼66% to ∼16% upon heating to 130 °C, where it is completely denatured,^63^ and the results of this study are in agreement. Therefore, we defined the denaturation temperature as the temperature at which the α-helix content becomes 41% (the midpoint between 66 and 16%). By linear fitting between 45 and 85 °C, the temperature at which an α-helix content of 41% was reached was determined to be ∼59 °C.

Figure 1c shows the results of BSA denaturation observed by DSC, where the temperature was increased and decreased three times between 25 and 85 °C. A broad endothermic peak was observed only during the first temperature increase. The thermal anomaly ranged from approx. 45 to 85 °C, but the peak was at ∼61 °C, which was chosen as the denaturation temperature. This is in good agreement with the CD spectrum results. The disappearance of the endothermic peak in the second and third temperature increases indicates that once denaturation occurs at elevated temperatures, the folded structure does not recover upon cooling,^63^ and the denatured state is maintained. From these results, the denaturation temperature of BSA in pure water was determined to be ∼60 °C.

### Hydration Changes Measured Using THz Spectroscopy

**Figure 2:**
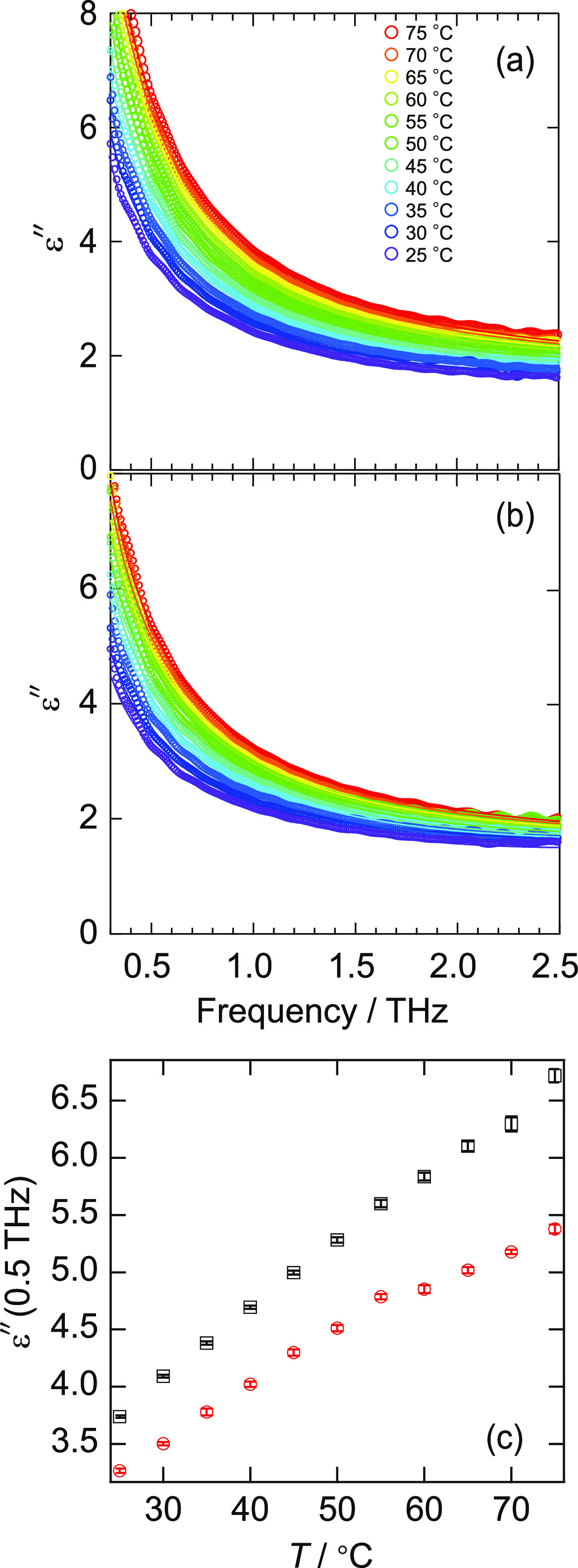
Results of THz spectroscopy. The imaginary part of the dielectric constant obtained by THz-TDS for (a) pure water and (b) BSA solution. The data shown were the averages of at least four times measurements for the same conditions. Solid lines are the fitting results by eq 3. (c) Imaginary part of the dielectric constant at 0.5 THz for pure water (□) and BSA solution (○). Error bars are 1σ of the multiple measurements at least four times.

THz spectroscopy was utilized to investigate the hydration state of BSA, and the results are shown in Figure 2. Figure 2a,b shows the imaginary parts of the complex dielectric constant ε″ of pure water and BSA solution in the THz frequency band, respectively. In both cases, the intensity of the spectra increased with increasing temperature. Three or four modes of water dynamics exist within this frequency band.^45−47^ The most significant contribution comes from the slow relaxation mode of the collective rotational relaxation of water, which peaks at ∼0.02 THz. The intensity of this relaxation is so large that it is still observed in the THz frequency band. The increase in the spectrum with increasing temperature is attributed to the fact that slow relaxation becomes faster, and the spectrum shifts to a higher frequency.^45,46^ In addition, a collisional relaxation mode of water exists at ∼1 THz (fast relaxation), corresponding to an isolated water molecule that is transiently broken off from the hydrogen-bonding network. Furthermore, intermolecular bending and stretching vibrational modes exist at ∼4 and 6 THz, respectively. As the intensity of the bending mode is very weak,^47^ it can be ignored for this investigation. The absorption by the protein in this frequency range is also weak enough to be ignored.^60,64^ In this study, we discuss the changes in the hydration state owing to slow relaxation. When water molecules are bound by a solute, the slow relaxation of the bound water shifts to the lower frequency side and is not observed in the THz range.^52^ Only the relaxation of the residual bulk-like water is observed. Here, hydration water is defined as water whose slow relaxation dynamics are not observed in the THz frequency band. Thus, the number of hydration water is determined by the degree of reduction in the intensity of the slow relaxation mode. Indeed, if we compare pure water and BSA solution, we can see that the intensity of the BSA solution is smaller than that of pure water. Figure 2c shows the ε″ at 0.5 THz for pure water and BSA solution. At this frequency, the slow relaxation mode dominates the spectrum, and the difference between water and BSA solutions reflects a decrease in the bulk-like water. These results indicate that the difference appears larger at higher temperatures than the denaturation temperature, implying that the number of hydration water is larger for the denatured state. Therefore, we considered the three modes (the slow and fast relaxation and stretching vibration) and fitted the spectrum with the following equation in the range of 0.3–2.5 THz^45−47,49^
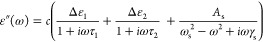
3*c* is the volume fraction of water in the system, and its value at each temperature was obtained from the density measurement of the BSA solution, as shown in Table S1. The first term in parentheses refers to slow relaxation, the second term is fast relaxation, and the third term is intermolecular stretching vibration. Δε_1_ and Δε_2_ are the strengths of the slow and fast relaxations, respectively. τ_1_ and τ_2_ are the relaxation times of their respective modes. *A*_s_, ω_s_, and γ_s_ are the amplitude, resonant angular frequency, and damping constant, respectively, of the intermolecular stretching vibration mode. For fitting to pure water, Δε_1_ and Δε_2_ were set as free parameters, whereas the other parameters were fixed as previously reported.^46^ We confirmed that the obtained Δε_1_ and Δε_2_, which dominantly affect the calculation of the hydration number, were in good agreement with previously reported results.^46^ To fit the BSA solution results, only Δε_1_ was used as a free parameter. Δε_2_ was fixed at 2.37, which was the value obtained by fitting the BSA solution result at 25 °C without fixing Δε_1_ and Δε_2_. The other parameters were fixed at the same values as those of pure water. The parameters obtained by fitting are shown in the Supporting Information for pure water and BSA solutions (Tables S2 and S3). As shown in Figure S2, Δε_1_ linearly decreased with the temperature for pure water, whereas it showed a relatively large gap of approximately 55–60 °C for the BSA solution.

Thus, we calculated the hydration number bound to BSA proteins using the following formula considering Kirkwood’s correlation factor (*g*_1_ = 2.9 for slow relaxation and *g*_2_ = 1.0 for fast relaxation) for these modes^49,65^
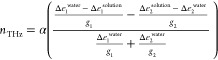
4The superscripts “water” and “solution” denote the results of pure water and BSA solutions, respectively. α is the total number of water molecules (bulk water and hydration water) per BSA molecule added to the solution (24,515 ± 112). Here, we assume that the slow and fast relaxations are isolated from each other, and the hydration water is estimated from the difference between the decrease in bulk-like water and the increase in fast relaxation. The hydration water estimated using THz spectroscopy is expected to be the sum of strong and weak hydration water.^53^

**Figure 3:**
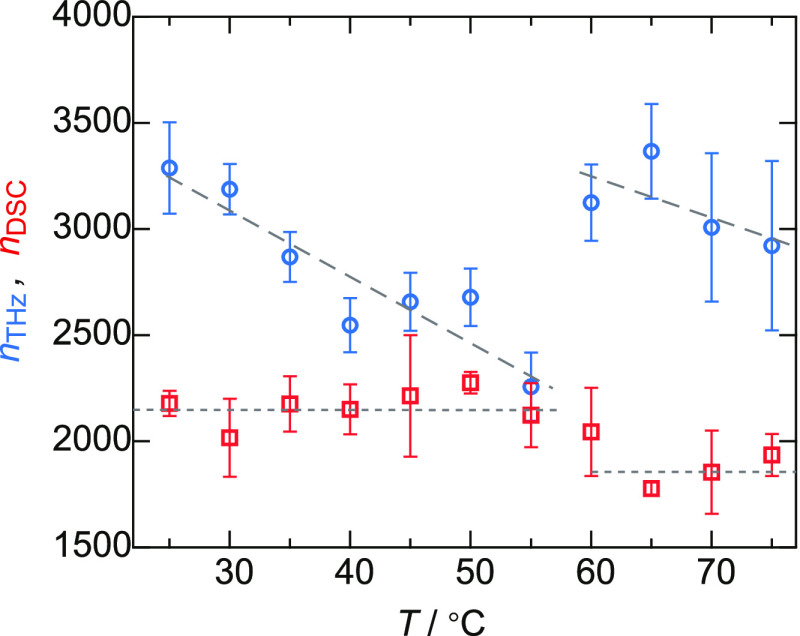
Obtained numbers of hydration water per BSA by THz-TDS (*n*_THz_, ○) and DSC (*n*_DSC_, □). Dashed and dotted lines are eye guides. The error bar for *n*_THz_ is calculated from the fitting errors of BSA solution and water and that for *n*_DSC_ is 1σ after performing measurements four times.

Figure 3 shows the obtained hydration number *n*_THz_ per BSA. At 25 °C, *n*_THz_ is ∼3300, which is comparable to the reported value for BSA and human serum albumin (HSA).^32,60^ The calculated number of hydration water molecules gradually decreased with temperature in the native state below 60 °C. This behavior indicates that native BSA is dehydrated with temperature, probably because of the activation of water dynamics. The calculated hydration number clearly shows a jump to a larger value at 60 °C, that is, by the denaturation of BSA, which is consistent with the reported results.^37^ It is expected that the bound water molecules around the exposed hydrophobic groups of the protein are newly observed as hydration water, which may be because of the hydrophobic hydration water.^36^ A greater number of hydration water at a partially hydrophobic surface than at a hydrophilic surface has also been reported for phospholipid bilayers.^50,57^

### Hydration Changes Measured by the DSC

**Figure 4:**
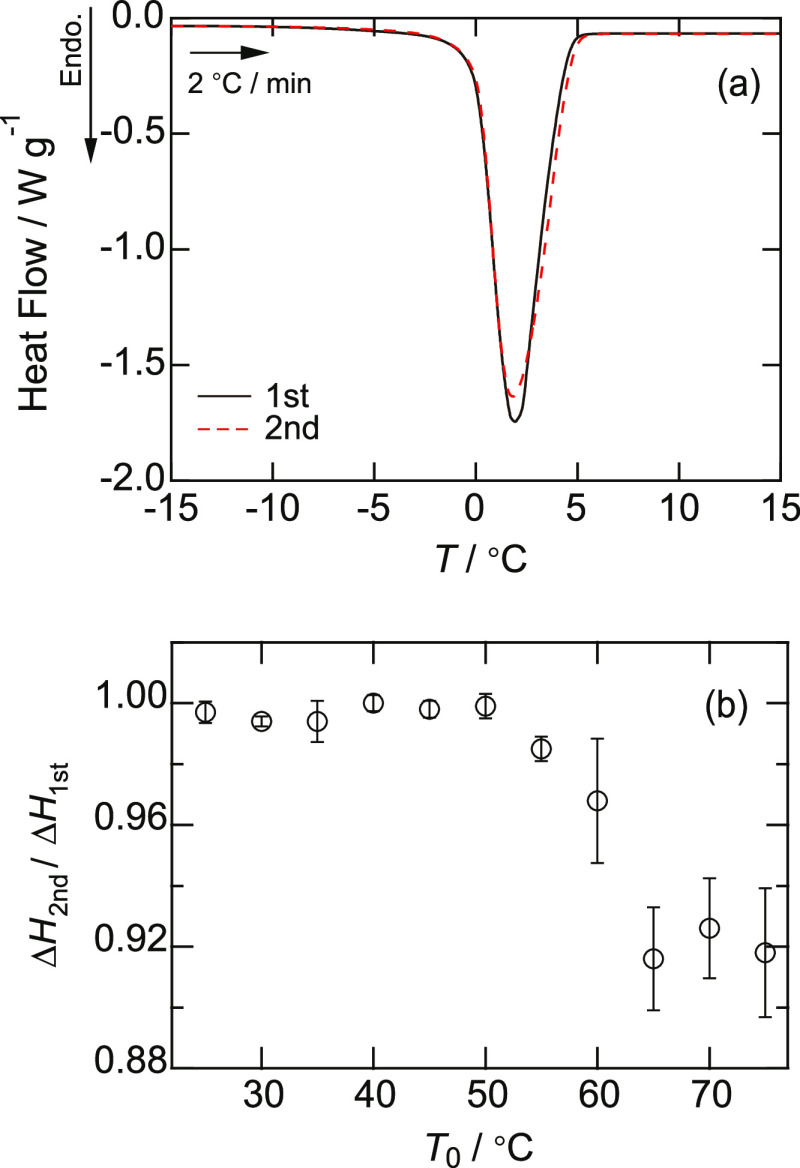
Results of DSC for determining unfreezable water. (a) DSC curves of BSA solution before (solid line) and after (dashed line) heating to 75 °C. (b) Ratio of the melting enthalpies of water during the first and second cycles. The error bar is 1σ after four times measurements.

The hydration of solutes has been widely investigated using thermal measurements and has predominantly focused on unfreezable water. Figure 4 shows the results of DSC measurements for the evaluation of unfreezable water.^4,38,39^ The thermal anomalies due to the melting of ice before (1st heating) and after (2nd heating) heating to 75 °C are shown in Figure 4a. The anomaly was smaller for the results obtained after heating above the denaturation temperature. As the BSA protein does not refold to its native state after denaturation, the thermal anomaly after heating is expected to reflect the freezable water in the denatured BSA solution. This indicates that the number of unfreezable water decreased by denaturation. The ratio of the transition enthalpy of water melting before and after heating to an arbitrary temperature, *T*_0_, is shown in Figure 4b. The ratio is almost 1 below the denaturation temperature, while it becomes 0.92 above the denaturation temperature.

We calculated the number of unfreezable water molecules per BSA as shown in Figure 3. Here, the number of unfreezable water at *T*_0_ is assumed to be the unfreezable water after heating to *T*_0_. In contrast to the change in the hydration number evaluated by THz spectroscopy, the number of unfreezable water molecules decreased with denaturation at 60 °C. When *T*_0_ < 60 °C, the protein may refold to some extent after cooling to 0 °C.^63^ In this case, the number cannot represent the exact number of unfreezable water molecules at *T*_0_, and it may represent only the number at 0 °C in the native state. However, when *T*_0_ > 60 °C, the proteins rarely show refolding.^63^ This indicates that the number of unfreezable water in the denatured state is less than the number at 0 °C. In other words, there is no doubt that the number of unfreezable water is smaller in the denatured state.

As addressed in the introduction section, the hydration water observed by THz spectroscopy and unfreezable water measured by DSC is completely different. THz spectroscopy detects hydration by the change in the water molecular dynamics in the ps timescale and evaluates hydration water, including both the strongly and weakly bound water (total of the first and second hydration shells). In contrast, unfreezable water is likely to only include strongly bound water. Indeed, below the denaturation temperature, the number of hydration water observed by THz spectroscopy was larger than that of unfreezable water. Therefore, it is unsurprising that the changes in the numbers due to denaturation are completely different. The origin of this contrasting behavior will be discussed later.

### Changes in Hydrogen Bonding Measured by IR Spectroscopy

**Figure 5:**
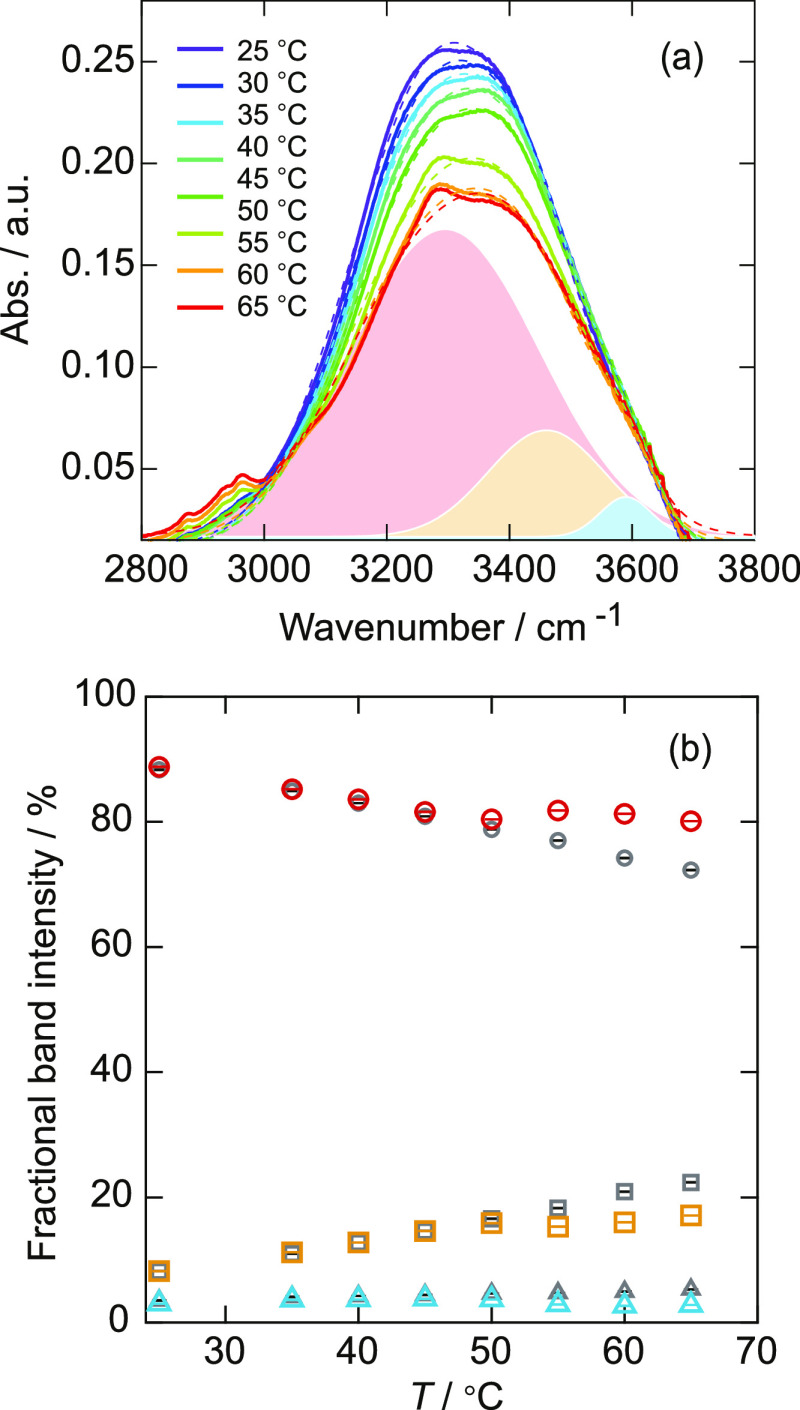
Results of FTIR spectroscopy of BSA solution in the OH stretching vibration region between 2800 and 3800 cm^–1^. (a) Solid lines are the measured results, dashed lines are the fitting results by three Gaussians, and the red, yellow, and blue filled peaks represent separate Gaussians for the results at 65 °C. (b) Fractional band intensities at 3295 (○), 3460 (□), and 3590 cm^–1^ (△) for pure water (black symbols) and BSA solutions (color symbols). Error bars within the symbols were calculated from the fitting errors.

Compared to THz spectroscopy and DSC, IR spectroscopy provides more microscopic information about hydration, that is, the hydrogen bonds between water molecules. It is known that the OH stretching vibration mode exists at 3000–3500 cm^–1^ depending on the strength of the hydrogen bond.^44^ When the OH group does not have (or only has very weak) hydrogen bonds with other molecules and exhibits almost free vibration, the vibration mode exists at 3590 cm^–1^. The vibration mode was at 3295 cm^–1^ for the strongly hydrogen-bonded OH group. In this case, the water molecule had nearly four hydrogen bonds with the other molecules. When the strength of the hydrogen bond is intermediate, and the number of hydrogen bonds per water molecule is 2–3, the band shifted to 3460 cm^–1^. These three vibrations coexisted in pure water: The strong hydrogen bond is ∼80%, the intermediate hydrogen bond is ∼15%, and the weak (or no) hydrogen bond is ∼5%. In this study, we investigated the change in the fraction of these three vibrations by changing the temperature of the BSA solution. Figure 5 shows the FTIR results of the BSA solution. To distinguish between the three vibrations, we performed functional fitting with three Gaussian functions and obtained the integrated intensity of each vibration mode, as exemplified in Figure 5. The calculated fractions of the three vibrations are shown in Figure 5b for pure water and BSA solution. For pure water, the fraction of the strong-hydrogen-bond OH decreased with temperature and those of the intermediate- and weak-hydrogen-bond OH increased. Below the denaturation temperature of 60 °C, the fractions in the BSA solution were similar to those in pure water. Differences were observed at the denaturation temperature as follows: the fraction of the strong-hydrogen-bond OH became larger than that for pure water, and both the intermediate- and weak-hydrogen-bond OH became smaller at this temperature. In the solution of denatured BSA, the strong-hydrogen-bond OH is increased when compared to pure water. These results indicate that the hydrogen-bond structure in water is slightly affected by the native BSA state, while it is strengthened by denatured BSA. The increase in the strong-hydrogen-bond OH likely corresponds to the results of THz spectroscopy; that is, the collective rotational dynamics of the water molecules are inhibited by the strong hydrogen bonds with the other water molecules that have been increased.

## Discussion

In this investigation, we employed three methods to evaluate the hydration state of BSA and its changes upon denaturation. As shown in Figures 3 and 5b, the results provide information regarding hydration from different viewpoints. The THz spectroscopy showed that the total number of strongly and weakly bound water molecules, respectively, were related to the collective rotational dynamics. In contrast, DSC measurements can be used to evaluate the number of unfreezable water, which likely corresponds to the strongly bound water. In comparison, IR spectroscopy provides information on the strength of hydrogen bonds among water molecules. We have assembled these results considering the structural changes in BSA by denaturation and discussed the different behaviors of strongly and weakly bound hydration water.

First, we discuss the hydration state of native BSA. In the native state, proteins generally form folded structures due to the chemical bonds between the polar groups in a protein, which helps avoid exposing the hydrophobic part to water. Hydration thus mainly occurs on the hydrophilic surface of BSA. The DSC results indicate that the strongly bound water, mainly in the first hydration shell, is ∼2000 water molecules per BSA molecule, corresponding to an average of two water layers at the surface.^66,67^ However, THz spectroscopy results indicated 2200–3300 bound water molecules at the surface when the number of strongly and weakly bound water was added. The extra 200–1300 hydration water molecules likely exist, mainly in the second hydration shell. Furthermore, a previous NMR study reported that BSA contained ∼30 strongly bound water molecules and ∼3000 weakly bound water molecules.^29^ The total number of strongly and weakly bound water was comparable between THz-TDS and NMR. The strongly bound water observed by NMR seems to be water that is directly bonded to functional groups,^30^ which moves very slowly, whereas the unfreezable water observed by DSC is likely to be water that is not directly bonded but whose movement is highly constrained through hydrogen bonds and electrostatic interactions. The number of weakly bound water molecules measured by THz spectroscopy decreases with temperature, and this is probably because of thermal activation, which is consistent with the results of IR spectroscopy, indicating a decrease in strong hydrogen bonds.

The responses of hydration to the denaturation of BSA are characteristic depending on the measurement methods. While the number of strongly bound water measured by DSC decreased with denaturation, the number of weakly bound water measured by THz spectroscopy increased. This indicates that the number of weakly bound water largely increases, accompanied by the denaturation of BSA. Considering that the folding structure is collapsed, and the inner core of the protein is exposed to water by denaturation, it is expected from the THz spectroscopy results that the newly hydrated hydrophobic part will mainly contribute to weakly bound water, which is in agreement with previous experiments and simulation results.^36,55^ This is also consistent with the hydration states of the phospholipid bilayers; a more hydrophobic surface has more weakly bound water than a more hydrophilic surface.^50,57^ This result is likely related to hydrophobic hydration.^55,56^ There are smaller number of weakly bound water in the native state than in the denatured state, which is consistent with the fact that many water molecules around an intrinsically disordered protein, which is mostly composed of hydrophilic polar groups, is strongly bound water.^68^ It is also possible that the polar groups in the protein that bind to each other in the native state are exposed to water in the denatured state, and that weakly bound water is detected around the exposed polar groups. With denaturation, the unfolded chains of the proteins become entangled with each other, and the protein solution forms a gel state.^15,19^ It is expected that the hydrophilic part that is exposed to water in the native state entangles with each other in places and that the part that interacts with water decreases. Thus, the number of strongly bound water was decreased because of denaturation. The FTIR results provided additional information. Upon denaturation, the strong hydrogen bonds of water were found to increase when compared to pure water, whereas the intermediate and weak hydrogen bonds decreased. This response was consistent with the THz spectroscopy results. That is, the strong hydrogen bonds increase, accompanied by an increase in the number of weakly bound water, indicating that the hydrogen bond in weakly bound water is stronger than that in pure water. The binding of water to each other by the hydrogen bonds is likely the cause of many weakly bound waters in the denatured state. The change in the hydrogen bond occurs at a slightly lower temperature (55 °C) than the change in the number of weakly bound water (60 °C), implying that an increase in the hydrogen bond antecedes the increase in the number of weakly bound water. It is noticed that the number of hydration water was implied to decrease upon pH-change-induced denaturation of a protein by THz spectroscopy,^69^ which is a contrasting trend to the present study. It is possible that the balance of polar and nonpolar groups in the protein was drastically changed by pH change, in contrast to the present study.

These results are related to the thermodynamics of hydration water reported in the literature.^20,21,26^ The partial enthalpy of hydration water is reported to be lower in the denatured state, which is the main cause of the relative destabilization of the native state of proteins. The entropy of water molecules is reduced by the denaturation. These thermodynamics have indicated that more water molecules are bound in the denatured state than in the native state, which is consistent with the THz spectroscopy result in the present study. Further, the present results imply that the decreased partial enthalpy of water is due to the increase in the stronger hydrogen bonds in the denatured state. Islam et al. experimentally reported that an increase in the hydrophobic residues at the surface of the protein leads to the stabilization of the protein through a decrease in the enthalpy of hydration water.^70^ Barnes also reported that more hydrophobic sites on the protein surface were hydrated, with slower diffusion of water.^71^ Sumi and Imamura reported by simulation that water drives the exposure of the hydrophobic groups of proteins, which is a critical factor for the destabilization of native state proteins.^72^ It has also been shown that the absorption of the THz wave is reduced as water molecules enter into the hydrophobic pockets of the protein, indicating that the hydrophobic part of the protein restricts the water dynamics.^73^ The present results are consistent with the findings of these reports; that is, the exposure of hydrophobic groups to water by denaturation increases the hydration water molecules with strong hydrogen bonds, leading to a decrease in the enthalpy in the denatured state. This increase in the number of hydration water is not explained by strongly bound water, indicating that weakly bound water plays a crucial role in the energy balance of the proteins. Because the layer of weakly bound water extends for several layers from the surface of the protein, it is suggested that water within the long range (as much as 1 nm) affects the stability of the protein.

The present findings are strongly related to the effects of osmolytes (small organic molecules) on protein stability. Recently, using THz spectroscopy, we reported that the effects of osmolytes on protein stability were dominated by a water-mediated indirect interaction rather than a direct interaction with osmolytes,^49^ which is consistent with the simulation study.^74^ The osmolytes change the dynamics of weakly bound water, which controls protein stability. This is consistent with the results of this investigation, as the states of weakly bound water were found to affect stability. How osmolytes alter weakly bound water on the protein surface is an interesting question that requires further research. Based on the present results, THz spectroscopy is expected to be a powerful tool for measuring weakly bound water with high-level accuracy.

Finally, we discuss the relationship between the hydration information observed using various methods. In this study, we conducted THz spectroscopy, thermal measurements, and IR spectroscopy to measure weakly bound water, strongly bound water, and hydrogen-bond strength, respectively. The results show that the behaviors of weakly bound water and strongly bound water are independent. This is because strongly bound water is mostly present around hydrophilic groups, while weakly bound water is also common around hydrophobic groups.^55^ However, there seems to be a correlation with weakly bound water in terms of hydrogen-bond strength. In other words, weakly bound water is thought to be caused by strong hydrogen bonds between water molecules. Based on the above discussion, the increase in the number of weakly bound water is due to the exposure of the hydrophobic groups to water. In other words, hydrogen bonds between water molecules around hydrophobic groups become stronger, resulting in weakly bound water. This is in good agreement with the picture of hydrophobic hydration that has long been proposed but is still a matter of dispute,^56^ i.e., the formation of ”iceberg” structures around hydrophobic functional groups.^55,75^ These results indicate that it is very important to understand the mobility and hydrogen-bonding structures of weakly and strongly bound water to accurately understand the hydration state of a material.

The results of this study will contribute to applied sciences, as it has recently been highlighted that controlling the water state is important for the development of smart (bio)materials.^4,6−14,39^ For example, here, we suggest that the control of the weakly bound hydration water is a key factor in the design of protein stabilizers, which may be valuable for food science. In other examples, the interactions between synthetic polymers and biomolecules, such as proteins, are closely related to the weakly bound hydration water (intermediate water), and its control is important for creating biomaterials for medical devices.^4,39^ It has also been reported that the lubrication of materials in water depends on the hydration state at the sub-nanometer scale.^8^ The present findings and methodology are important as we move forward with research on these applications, as they suggest that the design of the exposed hydrophobic groups is the key to controlling the hydration state, including the weakly bound hydration water at the sub-nanometer scale. As numerous unknowns regarding the state of the weakly bound hydration water in the second hydration shell at the sub-nanometer scale still exist, future detailed studies through experiments, theory, and simulations are expected to greatly advance our understanding of the physical properties of various materials in water and thus further the development of new industrial materials.

## Conclusions

In this study, we observed the changes in the hydration state of proteins upon denaturation using three measurement techniques that provide different information on hydration: THz spectroscopy, thermal measurement, and infrared spectroscopy. THz spectroscopy provides the combined hydration number of strongly bound water and weakly bound water, whereas thermal measurements only provide information regarding unfreezable water, that is, strongly bound water. These observations show completely different changes with denaturation. The number of strongly bound hydration water decreased with denaturation, while the number of weakly bound hydration water increased. When the results of IR spectroscopy were used, it was found that the strength of the hydrogen bonds among the weakly bound water became stronger, suggesting that the strengthening of the hydrogen bonds between water molecules reduces the overall motion of the water molecules. These results indicate that when the hydrophobic groups in the protein are exposed to water upon denaturation, more weakly bound water is created around them. This is thought to be caused by stronger hydrogen bonds between water molecules rather than between water and hydrophobic chains, which is likely related to hydrophobic hydration (iceberg-like formation). This result provides microscopic information on protein stability that has been explained thermodynamically, that is, that hydration relatively destabilizes the native state. The present results may also be related to the microscopic mechanism of recent reports that proteins become more stable as their hydrophobic surfaces increase.^70−72^ However, the entanglement of hydrophilic groups upon denaturation may reduce the number of sites where water can bind strongly, reducing the strongly bound hydration water. We could reveal that hydration information by each methodology does not necessarily change in the same way by external stimuli, and the integration of various types of information is important for universal understanding.

## Supporting Information Available

Density of pure water and BSA solutions and the fitting analysis of THz-TDS results (PDF)
