# Correction to “Contrasting Changes in Strongly and Weakly Bound Hydration Water of a Protein upon Denaturation”

**DOI:** 10.1021/acs.jpcb.4c02624

**Published:** 2024-05-07

**Authors:** Mafumi Hishida, Ayumi Kaneko, Yasuhisa Yamamura, Kazuya Saito

The data description of DSC results should be corrected. “The anomaly was smaller for the results obtained after heating above the denaturation temperature.” on page 6300 in the main text should be “Although the anomaly after heating above the denaturation temperature looks smaller, it is broader than that before the denaturation and the transition enthalpy is larger after denaturation.” Closeup of Figure 4(a) is Figure C1. Accordingly, the description of the vertical axis of Figure 4(b) should be corrected to Δ*H*_1*st*_/Δ*H*_2*nd*_ (Figure C2). Figure 3 and final conclusions do not change.

**Figure C1:**
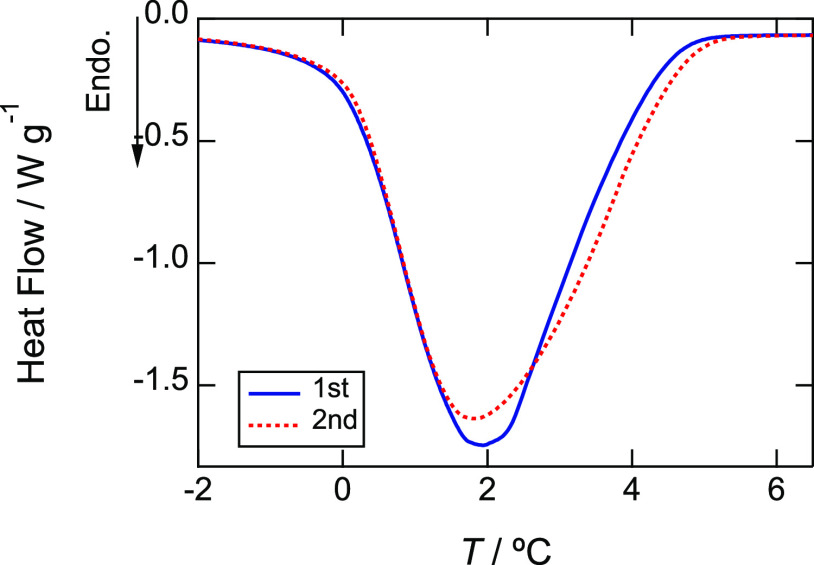
Closeup of Figure 4a in the main text.

**Figure C2:**
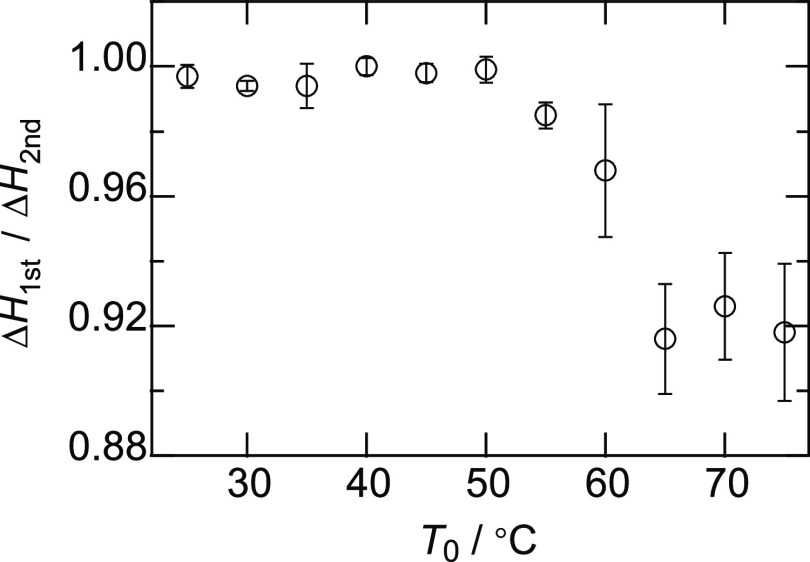
Corrected figure of Figure 4b in the main text.

