# Supplementary material for: Contrasting Changes in Strongly and Weakly Bound Hydration Water of a Protein upon Denaturation
Source: J Phys Chem B. 2023 Jul 7;127(28):6296–305. doi: 10.1021/acs.jpcb.3c02970 (PMC10364084; doi:10.1021/acs.jpcb.3c02970)
Supplement: Supplementary file 1 — jp3c02970_si_001.pdf [file jp3c02970_si_001.pdf]

Supporting Information

Contrasting Changes in the Strongly and Weakly Bound Hydration Water of a Protein Upon Denaturation

Mafumi Hishida,[1*] Ayumi Kaneko[2], Yasuhisa Yamamura[2], Kazuya Saito[2]

[1] Department of Chemistry, Faculty of Science, Tokyo University of Science, 1-3 Kagurazaka, Shinjuku, Tokyo 162-8601, Japan.
[2] Department of Chemistry, Faculty of Pure and Applied Sciences, University of Tsukuba, Tsukuba, Ibaraki 305-8571, Japan

*E-mail: hishida@rs.tus.ac.jp
*Phone: +81-3-5228-8252

The density of the pure water and BSA solutions

To obtain the volume fraction of water in the BSA solution, which was used to fit the terahertz time-domain spectroscopy results using Eq. (3) in the main text, the densities of the pure water and BSA solution were measured using a density meter (DMA 35, Anton Paar). Pure water or the BSA solution was first heated to 40 °C and then poured into a density meter. The density at each temperature was measured as the sample temperature gradually decreased. The results are shown in Fig. S1. We confirmed that the results for pure water were in good agreement with a previous study [S1].

As this measurement could only be performed below 40 °C with DMA 35, the density above this temperature was obtained by extrapolating the data below 40 °C. The extrapolation was conducted as follows. Fitting a quadratic function to the results for pure water yields $d_{\mathrm{water}}/\mathrm{g\,cm^{-3}} = -4\,T^2/10^{-6} - T/10^4 + 1.0364$, where d_{water} is the density. This function is in good agreement with the literature values for the density of pure water above 40 °C [S1], as shown in Fig. S1. Therefore, the quadratic function was also fitted to the BSA solution below 40 °C, and we obtained $d_{\mathrm{BSA}}/\mathrm{g\,cm^{-3}} = -3\,T^2/10^{-6} - T/10^4 + 1.002$. This function was used to calculate the density of the BSA solution at temperatures > 40 °C. Using the obtained density for pure water and the BSA solution, we calculated the volume fraction c of water in the

BSA solution (Table S1). We assumed that the density of water in the BSA solution was the same as that of pure water at each temperature. In the BSA solution, we assumed that the density of the solution in the native and denatured states follows the same tendency and is represented by the same formula.

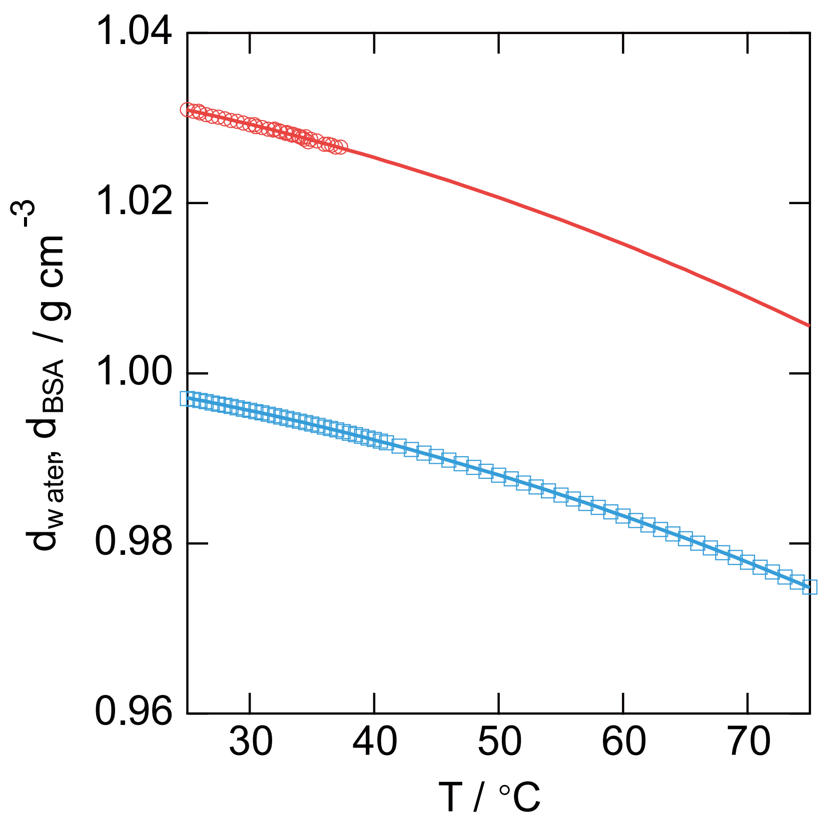

Figure S1: Densities of pure water d_{water} (blue square) and BSA solution d_{BSA} (red circle). The data for pure water > 40 °C are taken from the literature [S1]. Solid lines are the fitting results between 25 and 40 °C by quadratic functions.

Table S1: Volume fractions for the water in the BSA solution *c* [eq. (3) in the main text] obtained from the density measurement.

T / °C	*c*
25	0.8999
30	0.8997
35	0.8996
40	0.8995
45	0.8994
50	0.8993
55	0.8991
60	0.8989
65	0.8987
70	0.8984
75	0.898

Fitting the THz-TDS results

The imaginary part of the dielectric constant obtained using the THz-TDS was fitted using Eq. (3) (in the main text). The parameters were obtained for pure water, as described in the main text (the section "Hydration changes measured using THz spectroscopy"). To fit the BSA results, we fixed parameters other than $\Delta\varepsilon_1$ and $\Delta\varepsilon_2$ to those of pure water. $\Delta\varepsilon_2$ for the BSA solution was obtained by fitting the results at 25 °C. To precisely discuss the change in $\Delta\varepsilon_1$, we fixed $\Delta\varepsilon_2$ as the value at 25 °C (2.37). The obtained parameters are listed in Tables S2 (pure water) and S3 (BSA solution). The $\Delta\varepsilon_1$ for the pure water and BSA solutions are compared in Fig. S2. The values decreased almost linearly with the temperature for pure water, while the slope for the BSA solution appeared to change at approximately 60 °C, which is the denaturation temperature. The difference between $\Delta\varepsilon_1$ for the BSA solution and pure water corresponds to the amount of hydration water. These results indicate that the hydration state changes drastically at the denaturation temperature.

Table S2: Eq. (3) parameters obtained by fitting to the THz-TDS results for pure water.

T / °C	$\Delta\varepsilon_1$	τ_1 / ps	$\Delta\varepsilon_2$	τ_2 / ps	A_s / THz^2	ω_s / THz	γ_s / THz
25	72.4	8.30	1.82	0.252	31.2	5.27	5.41
30	71.3	7.37	1.83	0.258	31.0	5.24	5.64
35	69.9	6.59	1.79	0.263	30.9	5.21	5.88
40	68.8	5.94	1.76	0.269	30.7	5.18	6.12
45	67.3	5.38	1.74	0.274	30.5	5.15	6.35
50	65.7	4.90	1.76	0.280	30.3	5.12	6.59
55	63.9	4.48	1.78	0.285	30.1	5.09	6.82
60	61.8	4.12	1.82	0.291	29.9	5.06	7.06
65	59.8	3.80	1.84	0.296	29.7	5.03	7.29
70	57.7	3.52	1.86	0.302	29.6	5.00	7.53
75	55.9	3.27	1.89	0.307	29.4	4.97	7.76

Table S3: Eq. (3) parameters obtained by fitting the THz-TDS results for the BSA solutions.

T / °C	$\Delta\varepsilon_1$	τ_1 / ps	$\Delta\varepsilon_2$	τ_2 / ps	A_s / THz^2	ω_s / THz	γ_s / THz
25	60.4	8.30	2.37	0.252	31.2	5.27	5.41
30	59.8	7.37	2.37	0.258	31.0	5.24	5.64
35	59.4	6.59	2.37	0.263	30.9	5.21	5.88
40	59.3	5.94	2.37	0.269	30.7	5.18	6.12
45	57.6	5.38	2.37	0.274	30.5	5.15	6.35
50	55.8	4.90	2.37	0.280	30.3	5.12	6.59
55	56.2	4.48	2.37	0.285	30.1	5.09	6.82
60	51.6	4.12	2.37	0.291	29.9	5.06	7.06
65	49.3	3.80	2.37	0.296	29.7	5.03	7.29
70	48.5	3.52	2.37	0.302	29.6	5.00	7.53
75	47.2	3.27	2.37	0.307	29.4	4.97	7.76

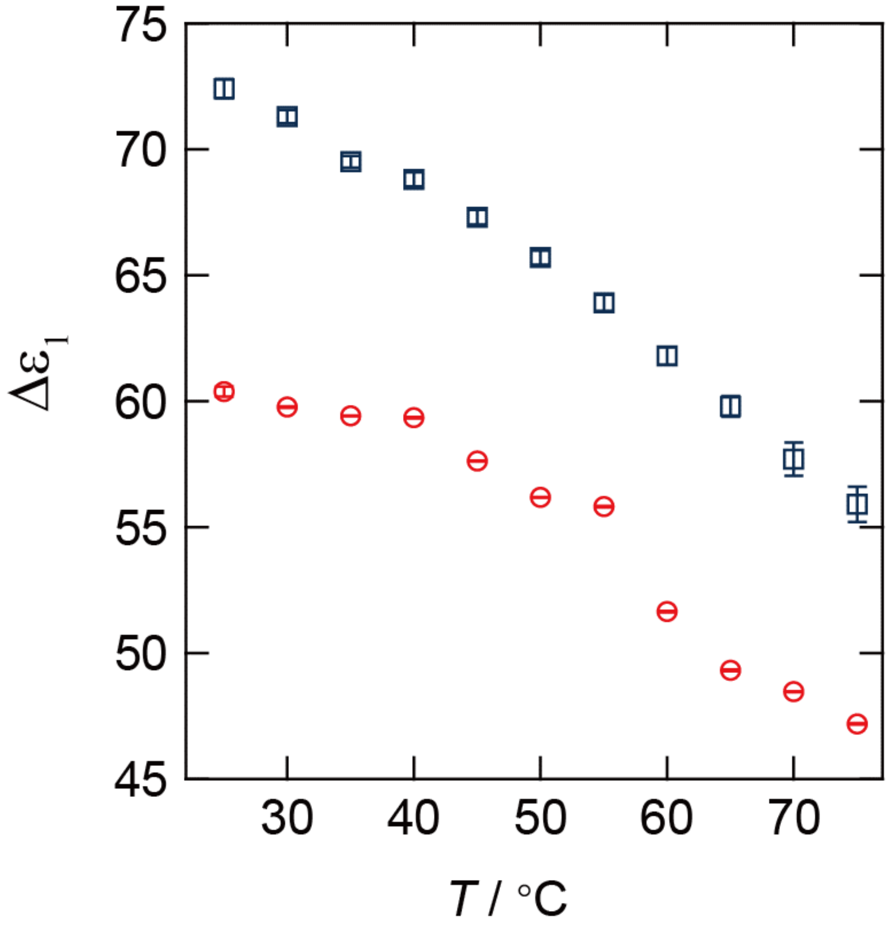

Figure S2: Obtained $\Delta\varepsilon_1$ for the pure water (square) and BSA solution (circle) at the specified temperatures. Error bars indicate the fitting errors by Eq.(3).

References

[S1] Kell G. S., Density, Thermal Expansivity, and Compressibility of Liquid Water From 0. deg. to 150. deg. Correlations and Tables for Atmospheric Pressure and Saturation Reviewed and Expressed on 1968 Temperature Scale. *J. Chem. Eng. Data,* **1975**, *20*, 97-106.
